# Strengths, challenges, and strategies for implementing pragmatic multicenter randomized controlled trials (RCTs): example of the Personalized Citizen Assistance for Social Participation (APIC) trial

**DOI:** 10.1186/s13063-024-08248-w

**Published:** 2024-06-27

**Authors:** Mélanie Levasseur, Agathe Chaintré-Prieur, Marie-France Dubois, Catherine Maisonneuve, Johanne Filiatrault, Helen-Maria Vassiliadis

**Affiliations:** 1 Faculty of Medicine and Health Sciences, Université de Sherbrooke, Sherbrooke, Canada; 2 Research Centre On Aging, Eastern Townships Integrated University Health and Social Services Centre – Sherbrooke University Hospital Centre, Sherbrooke, Canada; 3 Research Centre, University Institute of Geriatrics of Montreal (CRIUGM), Montréal, Canada; 4 Faculty of Medicine and Health Sciences, Université de Montréal, Montréal, Canada

**Keywords:** Random allocation, Health, Social engagement, Older adults, Facilitators, Barriers, Tactics, Community organization, Innovation, Multicenter collaboration

## Abstract

**Background:**

Randomized controlled trials (RCTs) are rigorous scientific research designs for evaluating intervention effectiveness. However, implementing RCTs in a real-world context is challenging. To develop strategies to improve its application, it is essential to understand the strengths and challenges of this design. This study thus aimed to explore the strengths, challenges, and strategies for improving the implementation of a pragmatic multicenter, prospective, two-arm RCT evaluating the effects of the Personalized Citizen Assistance for Social Participation (*Accompagnement-citoyen Personnalisé d’Intégration Communautaire*: APIC; weekly 3-h personalized stimulation sessions given by a trained volunteer over a 12-month period) on older adults’ health, social participation, and life satisfaction.

**Methods:**

A multiple case study was conducted with 14 participants, comprising one research assistant, seven coordinators, and six managers of six community organizations serving older adults, who implemented the APIC in the context of a RCT. Between 2017 and 2023, qualitative data were extracted from 24 group meetings, seven semi-directed interviews, emails exchanged with the research team, and one follow-up document.

**Results:**

Aged between 30 and 60 (median ± SIQR: 44.0 ± 6.3), most participants were women from organizations already offering social participation interventions for older adults and working with the public sector. Reported strengths of this RCT were its relevance in assessing an innovative intervention to support healthy aging, and the sharing of common goals, expertise, and strategies with community organizations. Challenges included difficulties recruiting older adults, resistance to potential control group assignments, design complexity, and efforts to mobilize and engage volunteers. The COVID-19 pandemic lockdown and health measures exacerbated challenges related to recruiting older adults and mobilizing volunteers and complicated delivery of the intervention. The strategies that mostly overcame difficulties in recruiting older adults were reducing sample size, simplifying recruitment procedures, emphasizing the health follow-up, extending partnerships, and recognizing and supporting volunteers better. Because of the lockdown and physical distancing measures, the intervention was also adapted for remote delivery, including via telephone or videoconferencing.

**Conclusion:**

Knowledge of the strengths and challenges of pragmatic RCTs can contribute to the development of strategies to facilitate implementation studies and better evaluate health and social participation interventions delivered under real-life conditions.

**Trial registration:**

NCT03161860; Pre-results. Registered on May 22, 2017.

**Supplementary Information:**

The online version contains supplementary material available at 10.1186/s13063-024-08248-w.

## Background

The number of people aged 65 and older totaled 0.7 billion worldwide in 2019 and is expected to reach 1.5 billion by 2050 [1]. Worldwide aging of populations and an increase in the number of people with complex health and social care needs generate both challenges [2] and opportunities for improving individual health and quality of life, and for allocating resources and ensuring equity in society [3]. Health and social services decision-makers and professionals need to implement effective interventions to maximize healthy aging, value older adults’ contributions, and help them integrate into society [4]. This has become even more crucial since the COVID-19 pandemic, which highlighted the importance of social participation in maintaining older adults’ health [5–7]. Relevant interventions are often delivered by nonprofit community organizations and cooperatives playing a vital role in healthy aging [8]. These community partners can innovate through various types of interventions that promote citizen involvement and an inclusive society. For example, during the COVID-19 lockdown, these partners informed older adults about public health instructions and offered them support and assistance [9]. To achieve the best interventions, community partners need guidance based on solid evidence [10]. However, very few interventions have been formally evaluated [11], especially those led by community organizations [12] or related to the social participation of older adults with disabilities [13].

Randomized controlled trials (RCTs) are considered the “gold standard” for measuring intervention effectiveness [14]. In addition to controlling many biases, RCTs contribute to the identification of possible causal associations [15]. Reports on RCTs of social and psychological interventions have, however, frequently been criticized due to concerns about their accuracy, comprehensiveness, and transparency [14]. These criticisms often revolve around the challenges of replicating these studies, assessing their findings, and gaining insights regarding for whom and under what circumstances these interventions should be delivered. Conducted in real-world settings, pragmatic RCTs are designed to reflect routine practice and how an intervention will be carried out in the community, making their results more generalizable to a broader population [16]. Challenges to conducting pragmatic RCTs aimed at evaluating social participation interventions in older adults [17–25] are often underreported. For example, most of these RCTs report significant dropout rates, similar to or larger than other studies, yet fail to provide strategies to limit them. Implementation is also complicated by the logistical challenges of conducting pragmatic RCTs across several organizations, which requires cooperation across different institutions and key actors. Because of its complexity, it is essential to have an advanced understanding of the strengths, challenges, and strategies of such an implementation. By evaluating the effectiveness of interventions rather than their efficacy under controlled experimental conditions, pragmatic RCTs are very valuable for sustaining real-world implementation while being more sensitive to societal constraints [26]. Recently, many pragmatic and multicenter RCTs had to be delayed or even terminated after the onset of the pandemic to ensure the safety of participants and to prioritize research on the prevention and treatment of COVID-19 [27–30]. Because of the unpredictable global situation, governments often advocated social distancing as a public health measure to prevent or slow the spread of the disease [29]. As a result, some clinical research teams had to halt recruitment [29, 30]. Since knowledge of COVID-19 and its prevention has improved, some clinical research trials have resumed, usually with protocol modifications [29, 30].

One of these pragmatic RCTs that has resumed is the Personalized Citizen Assistance for Social Participation (APIC; French acronym for *Accompagnement-citoyen Personnalisé d’Intégration Communautaire*) trial, which aimed to evaluate the intervention’s short- and long-term effects on older adults’ health, social participation, life satisfaction, and healthcare services utilization as well as its cost-effectiveness as delivered by six organizations [13]. The APIC is a promising intervention designed to foster the health and social participation of older adults with disabilities [31–34]. It involves a non-professional volunteer who, after 2–5 days of training, provides weekly stimulation sessions of 2–3 h over a period of 6–18 months targeting significant social and leisure activities that are otherwise difficult for older adults to do [33], such as joining a walking or physical activity group. In a previous pre-experimental design, the APIC was found to be feasible [35] when delivered by paid non-professional attendants who had 2 days of training and were supervised by a research team. Under these conditions, the APIC was found to increase older adults’ mobility, accomplishment of social activities, and frequency of leisure activities [33]. Complementing and extending professional healthcare services, the APIC helped older adults with disabilities resume, maintain, explore, and experiment with meaningful social activities [33]. The APIC also increased their psychological and physical well-being, feeling of control, connectedness, self-esteem, and motivation to accomplish activities. It was also shown to have benefits for the social participation of older adults with mental health issues [36] or visual impairments [34]. However, there is a need to further evaluate the APIC as delivered according to usual practices in Quebec (Canada), i.e., when volunteers are supervised by coordinators in community organizations. Currently, thousands of older Canadians receive friendly visits from volunteers, without being empowered to use their personal and environmental resources or being stimulated to participate in the community. Although it has proven difficult for both research and community key actors, disseminating and implementing scientifically supported interventions within communities is crucial to increase the availability of innovations that enhance health and living conditions [37]. To better scale up and enable organizations to sustainably integrate interventions into their practices, it is essential to focus on their implementation in real-life settings. The APIC-RCT [13] was launched in 2017 and faced certain challenges, including the COVID-19 pandemic, that are important to document. Using the example of this pragmatic multicenter RCT evaluating the effects of the APIC on older adults’ health, social participation, life satisfaction, and healthcare services utilization, the present study aimed to explore the strengths, challenges, and strategies for improving the implementation of this design.

## Methods

### Design

To achieve this objective, a multiple case study was used, i.e., a research design involving intensive examination of several similar but unique units in order to generalize the results [38, 39]. By enabling the use of multiple data gathering techniques that reinforce and confirm findings [40], multiple case studies are highly appropriate for in-depth investigation of complex real-life processes or activities [40], such as the APIC-RCT [13] in the present study.

### Data sources

#### Study context

This qualitative study was conducted as part of a larger randomized controlled trial to evaluate the short- and long-term effects of an innovative intervention (APIC) delivered by organizations on older adults’ health, social participation, life satisfaction, health services utilization, and cost-effectiveness. In this RCT, a total of 180 adults aged 60 or over, restricted in at least one instrumental activity of daily living and living in one of four large cities in the province of Quebec, Canada, were randomly assigned to the experimental or control group using a centralized computer-generated random number sequence procedure [13]. The experimental group received weekly 3-h personalized stimulation sessions delivered by a trained volunteer for the first 12 months. The sessions encouraged empowerment, gradual mobilization of personal and environmental resources, and community integration. The control group received the publicly funded universal healthcare services available to all Quebecers. Over 2 years (baseline and 12, 18, and 24 months later), self-administered questionnaires assessed physical and mental health (primary outcome; version 2 of the 36-item Short-Form Health Survey, converted to SF-6D utility scores for quality-adjusted life years), social participation (Social Participation Scale), and life satisfaction (Life Satisfaction Index-Z). Healthcare services utilization was recorded, and the cost of each intervention calculated.

#### Definition of cases

The cases were six organizations which implemented the APIC as part of the RCT, i.e., five nonprofit (three volunteer centers and two community organizations providing services for older adults) and one cooperative, and the research assistant who coordinated the implementation in collaboration with each organization.

### Participants

The participants in this study included everyone responsible for implementing the APIC-RCT (implementation team). One research assistant, seven coordinators, and six managers from six community organizations participated in the study. As they were in transition for this position, two coordinators from the same organization participated. The research assistant was responsible for the overall management of the APIC-RCT and for helping the organizations to implement it. In each organization, one coordinator was responsible for its implementation and was trained by the research assistant to recruit older adults, enroll volunteers, assign older adults to volunteers, gather informed consent, supervise baseline testing, train the volunteers, and coordinate the intervention (APIC). The managers supported their coordinators, motivated their team, promoted the APIC within their organization and its network, and managed their part of the budget for the APIC-RCT. The research assistant, managers, and coordinators also participated in the interdisciplinary committee to ensure the APIC-RCT progressed as planned.

### Data collection

To document the implementation of the APIC-RCT, mainly qualitative data from varying sources were collected between September 2017 and June 2023. With the health measures in place, the COVID-19 pandemic had an impact on implementation (see Results) but not on the study’s data collection timeline. First, one discussion-guided meeting led by the principal investigator and with all participants from the community organizations, together with the research team, identified the strengths and challenges of the APIC-RCT as well as strategies to improve its application. Seven individual semi-directed interviews were then conducted by four occupational therapy trainees, who were members of the research team and whose master’s project was on the implementation of the APIC-RCT [41]. The first interview was with the research assistant, followed by five with six coordinators, and one with a manager. One of these interviews was conducted with the two coordinators from the same organization that, as mentioned, were in transition for this position. One of the coordinators could not be interviewed but participated in the group meeting.

Two semi-structured interview guides were used: one version for the research assistant, and the other for the coordinators and manager. The research assistant’s guide included questions about her motivation to participate in the project, experience, and role with community organizations, volunteers, and older adults in implementing the APIC-RCT. The coordinator and manager interview guide included questions such as: “Tell me about your organization’s implementation of the APIC” and “How has the research project influenced the participation of older adults?” To ensure sufficient experience with the research design, the interviews were conducted at least 9 months after implementation of the APIC-RCT had started. These interviews, as well as the meeting, were recorded on digital audiotape and transcribed (verbatim). Between September 2017 and June 2023, implementation was regularly monitored by the research team to gather as much information as possible about it. During this period, 18 additional meetings with the coordinators or managers, and 5 interdisciplinary committee sessions were recorded, transcribed, and summarized. Emails exchanged with research team, reports (meeting highlights) validated by the implementation team, and one follow-up document were collected in addition to the research team’s memos (field notes on thoughts and observations). Finally, to describe the participants and context in which the APIC-RCT was implemented, a sociodemographic questionnaire was completed by the research assistant, coordinators and managers.

### Data analysis

Descriptive statistics were used to describe the participants (medians [m] and semi-interquartile ranges [SIQR] for continuous variables, frequencies [n] and percentages [%] for categorical variables). To document strengths, challenges, and strategies for implementing the APIC-RCT, a thematic content analysis of reports of meetings, transcripts of interviews, emails, memos, and a follow-up document was carried out [42]. The four occupational therapy trainees who were members of the research team first coded the data using a mixed coding grid, *i.e.*, an initial grid based on the Consolidated Framework for Implementation Research (CFIR) [43, 44] to which new themes emerging from the data were added. Developed by Damschroder and colleagues in 2009 [43] and updated in 2022 [44], the CFIR comprises five domains influencing implementation of an ‘innovation’, such as the APIC-RCT: 1) the innovation domain, 2) its outer setting, 3) its inner setting, 4) the characteristics of the individuals involved in the innovation and its implementation, and 5) the implementation process. To ensure credibility, reliability, and confirmability [45], about half the transcripts were co-coded by a research assistant and the principal investigator. Any discrepancies in coding were discussed with the research team until consensus was reached. Initial codes emerging from the content of the interviews were subsequently organized and labeled as strengths or challenges of the APIC-RCT, in addition to strategies used to improve its application.

## Results

### Participants

Aged between 30 and 60 (median ± SIQR: 44.0 ± 6.3), most participants were women (Table 1). While the research assistant and managers had about 10 years’ experience both in their positions and working with older adults, coordinators were relatively new in their role but had about 4 years’ experience with older adults. Managers had more years’ experience in implementing interventions than coordinators and almost all the participants had some university education (Table 1). Providing services to French-speaking older adults, the six organizations had 3 to 17 employees, 20 to 943 volunteers, and 330 to 759 clients (Table 2). Most of the organizations already offered social participation interventions for older adults and were working within the public sector.

**Table 1:** Characteristics of participants (*n* = 14)

	**Coordinators (** ***n*** **= 7)**	**Managers (** ***n*** **= 6)**	**Research assistant (** ***n* = 1)**
**Continuous variables**	**Median (SIQR)**		
Age (years)	47.0 (8.3)	43.0 (3.5)	34
Current job experience (years)	1.0 (1.3)	10.0 (4.4)	8
Experience in community settings (years)	20.0 (7.5)	19.0 (2.1)	-
Experience with older adults (years)	4.0 (4.8)	10.5 (5.0)	11
Experience in implementing interventions (years)	2.5 (2.1)	15.0 (2.0)	-
**Categorical variables**	***n*** ** (%)**		
Women	7 (100.0)	4 (66.7)	1
***Education***			
College	-	1 (16.7)	-
Undergraduate university program	4 (57.1)	2 (33.3)	-
Graduate university program	3 (42.9)	3 (50.0)	1

*SIQR* semi-interquartile ranges

**Table 2:** Characteristics of organizations (*n* = 6)

**Continuous variables**	**Median (SIQR)**
***Composition***	
Employees (#)	6.5 (1.3)
Volunteers (#)	265.0 (117.5)
Clients (#)	428.5 (79.1)
**Categorical variables**	**n (%)**
***Clients***	
Children	2 (33.3)
Teenagers	2 (33.3)
Adults	4 (66.7)
Older adults	6 (100.0)
***Services offered***	
To improve older adults’ social participation	4 (66.7)
In French	6 (100.0)
Collaboration with public sector	5 (83.3)

*SIQR* semi-interquartile ranges

Implementation of this large-scale APIC-RCT (six organizations in four different cities across Quebec, Canada) began in 2017 and, although recruitment of participants is completed, data collection is ongoing. The APIC-RCT’s challenges were amplified by and combined with those of the COVID-19 pandemic. Organizations had to react quickly to this exceptional situation to adapt their services and pursue their activities as much as possible while continuing to implement the APIC-RCT. The public health measures and physical distancing imposed by the government in March 2020 to protect vulnerable people, including volunteers and older adults targeted by this study, had a considerable impact on organizations’ operations. As most of them were aged 65 or over, volunteers had to provide the intervention virtually. This situation highlighted the lack of access to technology and limited digital skills of some volunteers and coordinators in the collaborating organizations. For about 2 years and with the support of the research team, the organizations had to adapt to telework, including using virtual communication tools and providing volunteers and participating older adults with support and digital training to continue implementing the APIC-RCT despite the pandemic.

### Strengths

Strengths were identified in all CFIR domains, but mostly in the inner setting, and also in the characteristics of the individuals and the implementation process. Among the *characteristics of the innovation*, the APIC-RCT was recognized for the quality of its research design and its contribution to supporting healthy aging in a society undergoing demographic changes. The added value and relevance of the APIC was also acknowledged, as expressed by one manager (ED): “I’m proud to participate in this research and I hope it makes a difference.” (Table 3). Even before their partnership, the research team and all the community organizations had already been engaged in thinking about how to promote active and healthy aging better (Table 3), as reported by the research assistant (RA): “What’s really helpful about the organizations that decided to implement the APIC-RCT is that they believe in [the intervention]. They know that there is a need in their community and that makes sense to them.”. Despite its complexity (see next section), the APIC-RCT was adapted to meet the diverse needs of older adults and organizations. This was achieved by adjusting the number of older adults to recruit, promotional strategies, and volunteer training, and by expanding partnerships, as summed up in the group meeting: “Throughout the project, it was really about adaptation.”

**Table 3:** Strengths ( +) and challenges ( −) of the implementation of the APIC-RCT

**Characteristics of the innovation (APIC-RCT)**
**Relative advantage**
Support of the APIC for healthy aging ( +)
Added value and relevance of the APIC ( +)
**Adaptability**
Meeting the diverse needs of older adults and organizations^a^ (+ / −)
**Complexity**
Recruitment of older adults in the context of a pandemic^a^ ( −)
Synchronized recruitment of older adults and volunteers to be paired according to common interests^a^ ( −)
**Design**
RCT (+ / −)
Duration of the volunteers’ training on the APIC^a^ ( −)
**Outer setting**
**Partnerships and connections**
Openness and support from community partners^a^ (+ / −)
**Inner setting**
**Structural characteristics**
Needs already identified and targeted by organizations ( +)
Exchanges of expertise, strategies, knowledge, and materials ( +)
Expertise in managing volunteers ( +)
Community knowledge ( +)
Coordinator turnover ( −)
**Relational connections**
Teamwork within organizations ( +)
**Culture — ** ***learning-centeredness***
Support from the research team ( +)
Support from managers ( +)
Support and reassurance for volunteers ( +)
**Compatibility**
Objective of the APIC-RCT consistent with the mission of all organizations ( +)
Alignment between the APIC and older adults’ needs ( +)
**Relative priority**
Having a common goal: success of the APIC ( +)
**Mission alignment**
Strong commitment of coordinators in the APIC ( +)
**Available resources**
Financial and human resources^a^ (+ / −)
**Access to knowledge and information**
Training content ( +)
Promotional tools, recruitment documents, and forms (+ / −)
**Characteristics of the individuals**
**Opinion leaders**
Positive attitude ( +)
**Capability**
Belief in the APIC ( +)
Understanding of the APIC-RCT by coordinators, older adults, and volunteers (+ / −)
Resistance of potential older adult participants to being assigned to the control group ( −)
**Motivation**
Motivation from coordinators, managers, and volunteers ( +)
**Implementation process**
**Planning**
Anticipation of coordinators’ workload^a^ (+ / −)
Underestimated and unanticipated difficulties^a^ ( −)
➢ Interesting and recruiting more isolated older adults
➢ Mobilizing and engaging volunteers
**Engaging — ** ***innovation deliverers***
Use of different promotional strategies ( +)
Identification of target older adult participants by community partners (+ / −)

Text in bold refers to concepts in Damschroder and colleagues’ CFIR [44]

^a^Challenges exacerbated by the COVID-19 pandemic

Within *inner settings*, organizations that had already identified and targeted the needs of older adults facilitated implementation of the APIC-RCT (Table 3). Collaboration was strengthened by regular and rich exchanges of expertise, strategies, knowledge, and materials between organizations and the research team, as one coordinator noted I: “The APIC gives us an interesting structure and guidelines for developing a new service, which can really help to maintain and improve the independence of [older adults].” (C4). The support offered by the research team and the provision of documents to guide volunteers were also appreciated: “When I look at the training and its content, I think it is ‘A1’. It is clear.” (C2). Moreover, the expertise of the volunteer managers, including their support, reassurance, and knowledge of the community, were major facilitators of the APIC-RCT implementation. The implementation was also enhanced by teamwork that was particularly well established within the organizations, as detailed by one coordinator: “We don’t always have time for individuals but we look at the team and say: ‘There’ll be four of us to manage it’. Two looked after training and volunteer recruitment, and two looked after older adults. We thought it’d be easier to manage if we split it up that way.” (C3). In addition, the objective of the APIC-RCT, i.e., advancing knowledge about the effects of health and social participation interventions on older adults, was aligned with the organizations’ values and mission (Table 3). Recognition by the organizations of the APIC’s relevance and alignment between this intervention and the needs of older adults strengthened the support from managers. This recognition was accompanied by the coordinators’ strong commitment and common goal of successfully implementing the APIC, as reported by one coordinator: “The project was really in line with everything we do: interventions helping people to be more independent and manage to remain independent for as long as possible. […] We believe in the project.” (C1). Such shared objectives fostered effective and valued collaboration between participants.

Belief in the APIC’s relevance and motivation from the coordinators and managers, as well as from volunteers, were among the *individual characteristics* that were the most decisive factors in the success of the APIC-RCT (Table 3). These characteristics facilitated the development of strategies to continue engagement in the project and overcome the difficulties, as explained in the group meeting: “This project fits in with the philosophy we want to implement, we’re really working to keep people in the community, so I’m keen to invest.” (M). Because of their positive attitude, organizations were willing to adapt and use various promotional strategies to ensure the success of the APIC-RCT (e.g., contact retirement homes, libraries, churches, community groups, issue a newsletter), as one coordinator explained: “Whether you’re Protestant, Anglican or whatever, you might need help! I started going to churches as a way of making contact. I got four reverends, four pastors who believe in the project, and it was very well received, without getting involved in religion.” (C4). This marked their commitment throughout implementation of the APIC-RCT.

### Challenges

Also found in all domains, the challenges encountered were mainly related to the characteristics of the innovation. Additionally, difficulties with the inner setting, the individuals, and the implementation process itself were identified (Table 3). Regarding the *characteristics of the innovation*, especially its complexity and research design, the recruitment of older adults was one of the major challenges in this study. Due to randomization and the risk of being assigned to the control group, some older adults were unwilling to participate in the project or dropped out, and many health and community partners referred fewer potential older adult participants to the study than expected (Table 3). Despite her years of experience with older adults and the fact that her organization had existed for over 35 years and had over 450 older adult clients, one coordinator brought up the recruitment challenge: “I met older adults who could have benefited from the intervention but didn’t want to participate because of the random assignment. The randomization was a real problem [and] created some ethical dilemmas [very isolated older adults randomly assigned to the control group while others who had a larger social circle benefited from the APIC].” (C2). Although the control group had access to the APIC at the end of the study, the 24-month wait time was considered too long.

The synchronization of recruitment also required a lot of time and effort on the part of coordinators (Table 3), as explained by this participant: “If we recruit volunteers at different times than [older adults], it doesn’t work. Both must be recruited at the same time*.*” (C6). Another coordinator also noted that this synchronization was further complicated by matching dyads according to common interests: “I had a nice sheet of questions for the first meeting of APIC volunteer I…] but when I got [only] two volunteers and then two older adults, I’m stuck with that.” (C2). Considered by the organizations as being too long (Table 3), the training of volunteers also added to the complexity in synchronizing dyad formation.

Regarding the *outer setting*, the complexity of the research design also restricted the support of partners outside organizations implementing the APIC-RCT, as expressed by a coordinator from the cooperative: “Community partners thought the APIC was appropriate for [older adults]. But it was hard to find a way to get them on board because there was some mistrust, and it wasn’t easy for everyone to understand what the APIC was.” (C2). Although mostly a facilitator, the *inner setting*, and especially the precariousness of the organizations’ financial and human resources, led to turnover among coordinators, which was a major challenge. Because of this turnover, they were not always able to devote as much time as desired to recruitment, which limited the human resources available (Table 3), as reported by one coordinator: “[A member of the team] left […] and was not replaced. That also had an impact on implementation.” (C7). The recruitment strategy was also not as effective as anticipated. Mass marketing (posters, media, etc.) was unsuccessful and, at the beginning, the promotional materials were too technical for the general population to understand.

In terms of the *characteristics of the individuals* involved, even though the research team, coordinators, and managers had some higher education, understanding the APIC-RCT was a challenge. Despite the availability of the research team, the complexity of the research design remained a barrier, as explained by this coordinator (Table 3): “Not everyone has taken a methodology course. We don’t understand everything that goes on behind a research project, it’s difficult.” (C5). The documentation pertaining to the study did not consider this issue enough. For example, the initial consent form presented complex information and required a signature from participating older adults, which made them wary of the research. Despite further adaptations and explanations about the study design, older adults’ resistance to being assigned to the control group persisted, making recruitment and subsequent participation particularly challenging, as described by one coordinator: “[Some older adults] who were in the control group wanted to drop out, didn’t want to participate.” (C6).

Finally, the *implementation process* was marked by unanticipated difficulties related to the coordinators’ workload. Because of their limited use of community and health services, some very isolated older adults were not known to the organizations and were hard to reach. The research team and the organizations had not anticipated the extent of recruitment difficulties, as one coordinator explained: “Maybe we were ‘arrogant’ but I think all the sites felt the same. We really thought we would recruit older adults easily.” (C4). Coordinators finally took an individual approach using lists of potential participants provided by Medical Records departments. Despite this strategy, coordinators had to contact many older adults before finding people interested in and eligible for the project. In fact, some potential older adult participants targeted by community partners were not eligible because of the exclusion criteria, *i.e.,* they had severe cognitive disorders or significant physical disabilities (for example, were unable to get out of bed without help). Other older adults refused as they were already socially active or had a good social network. In some cases, the recruitment took place at an inopportune time (summer or winter, illness, bereavement, etc.). Beyond the challenge of contacting isolated older adults, mobilizing and engaging volunteers was also a major challenge for the organizations, as explained by one coordinator: “It’s not surprising that we had difficulty recruiting volunteers, all community partners have this difficulty. Those who don’t have a problem are those with a very strong sense of belonging.” (C8). Sometimes, long-term involvement militated against engagement by the volunteers, particularly the younger ones, as highlighted by the same coordinator: “The new generation of volunteers don’t have the same mentality. They are more demanding, have other realities […]. The APIC is the opposite of this new reality since it asks for a 1-year commitment of 3 h a week.” (C8).

Some of these challenges were exacerbated by the COVID pandemic, as reported by one coordinator: “During the implementation, the pandemic didn’t make our work any easier. With the lockdown, we had the most complex clients. It wasn’t easy.” (C2). Sometimes, face-to-face assistance was not possible: for example, some environments were too small for proper physical distancing, volunteers were older adults themselves and, as such, were restricted, and seniors’ residences did not allow any visitors. As a result of all these challenges and to increase the number of participants, another organization was included 26 months into the project and the recruitment period was extended to 4 years and 10 months.

### Strategies

Most of the strategies focused on one of the *characteristics of the innovation*: recruiting older adults (Table 4). Organizations initially had to recruit a total of 376 older adults but, for feasibility reasons and while still considering the dropout rate and a larger effect size, this target sample size was reduced to 180. This reduction gave organizations a more realistic objective, especially during the pandemic. To reduce reluctance related to randomization, the participating older adults were all able to benefit from usual services and the APIC either during (for the experimental group) or after their participation in the intervention (for the control group). Another strategy was to present the APIC-RCT to older adults as a health follow-up with a 50% chance of getting the intervention (Table 4). All the participating older adults were interviewed by phone every 2 months to gather information about their use of health services. In addition to these interviews, they completed the same questionnaires on their health, social participation, and life satisfaction at 0, 12, 18, and 24 months after their enrolment. During the pandemic, as most of the volunteers were older adults themselves, with higher vulnerability to COVID-19, the organizations helped them deliver the APIC via long-distance communications, i.e., by phone or using a videoconference platform, depending on what equipment was available and the readiness and technical skills of the volunteers and older adults (Table 4). As one manager said: “Meetings often took place by phone. We had adapted them like that but it was quite a headache.” (ED). In addition to encouraging volunteers to continue their involvement from home, this period was also an opportunity to reflect on strategies to recruit younger volunteers through, for example, the volunteer bank created during the pandemic by the provincial government [46], and to develop intergenerational pairings. The research team and coordinators worked together to adopt strategies to reduce the impacts of the pandemic (i.e., higher risk of isolation, inability to meet face-to-face reducing motivation regarding the APIC, greater potential for attrition).

**Table 4:** Strategies to overcome challenges in implementing the APIC-RCT

**Characteristics of the innovation (APIC-RCT)**
**Complexity**
Supporting long-distance communications
Recruiting younger volunteers and increasing intergenerational pairings
**Design**
Reducing sample size
Receiving usual services and the APIC (during or after participation in the intervention)
Presenting the APIC-RCT to older adults as a health follow-up
**Outer setting**
**Partnerships and connections**
Extending partnerships with other organizations
**Inner setting**
**Available resources**
Developing volunteers’ sense of belonging, valuing their role and recognizing their commitment
**Characteristics of the individuals**
**Capability**
Enhancing coordinators’, older adults’, and volunteers’ understanding of the APIC-RCT by simplifying recruitment documents and forms
**Implementation process**
**Planning**
Improving volunteers’ mobilization and keeping them motivated

Text in bold refers to concepts in Damschroder and colleagues’ CFIR [44]

To increase recruitment opportunities, the partnership was extended to other organizations with different profiles, in the *outer setting* of the APIC-RCT. Although varying from one setting to another, new collaborations increased the number and diversity of people interested in participating who were recruited. In the *inner setting*, providing training in small groups, weekly personalized follow-ups with the coordinator, and monthly peer meetings helped volunteers to develop a sense of belonging and have their role valued and their commitment recognized, as stressed by one manager: “We know and appreciate what they do and it is exceptional. Recognition is really important. They are also taking part in a research study. They are certainly happy. It makes a difference.” (ED).

Furthermore, to make the project easier to understand in terms of the *characteristics of the individuals* involved in delivering the APIC-RCT, recruitment documents and forms were simplified. This facilitated the recruitment process for both coordinators and older adults (Table 4), as explained by the research assistant: “We drew up an information and consent form but in leaflet form, with a much simpler choice of words, closer to everyday language, and the ethics committee was very open to it. There were three documents: the leaflet, a form for older adults to sign, and another form for the signature of the person obtaining consent. It was less frightening than a 6-page document.” (RA). Finally, with regard to the *implementation process*, since the APIC-RCT requires a significant year-long commitment from volunteers, strategies were identified to improve the mobilization of volunteers and keep them motivated during the entire intervention (Table 4).

## Discussion

Using the example of the APIC-RCT, this study explored the strengths, challenges, and strategies for improving the implementation of a pragmatic multicenter, prospective, two-arm RCT evaluating the effects of the Personalized Citizen Assistance for Social Participation on older adults’ health, social participation, and life satisfaction. The strengths of the APIC-RCT primarily concern redesigning an innovative way to support healthy aging, with common goals and sharing expertise and strategies. Recruitment difficulties, potential older adult participants’ resistance to being assigned to a control group, the complexity of the research design, and efforts made to mobilize and engage volunteers also emerged as major challenges. Some of these challenges were exacerbated by the COVID-19 lockdown and health measures related to the pandemic.

Although recognized for their scientific rigor, RCTs used in a real-world context have been found to involve significant challenges [47, 48], especially in the case of social participation interventions for older adults like the APIC. To address these challenges, research strategies must be adjusted throughout the project [49, 50]. Adjustments made during the implementation phase play a crucial role in the process of scaling up an intervention [51]. In the study by Elliott and colleagues [47], organizational difficulties and organizations’ lack of time were major barriers to recruiting participants. Intellectual and emotional challenges in joining a research study with people working in the field, particularly given the complexity of the RCT’s design and the principle of randomization, were also previously observed [47]. Because these challenges were identified in the APIC-RCT, the research team was able to help coordinators to familiarize themselves with RCTs. As recommended by Elliott and colleagues [47], it is important to recognize the difficulties in order to see the benefit of assigning isolated older adults to the control group. Regular communications, shared values, and a common goal were also identified in an action research conducted in partnership with the community [52], as important and central strengths that help organizations in RCTs to adapt to both methodological constraints and ethical issues [48]. In the present study, coordinators ensured that older adults in the control group were offered services currently in place, including *Friendly visits*, an intervention that gave isolated older adults some companionship. Moreover, it was essential for all partners, including private seniors’ residences, to mobilize and collaborate in order to reach isolated older adults, as was also highlighted in a previous action research [52]. Consistent with the realist approach underlying this study, interventions do not work everywhere for everyone, and contextual factors (e.g., openness from community partners, support from managers, human resources) can make a difference [53].

The onset of the COVID-19 pandemic during implementation of the APIC-RCT also created new challenges. During this period, a population survey found that two in five older Canadians were worried about maintaining their social ties [54], especially because of restrictive health measures in place for vulnerable people [46]. Lockdown during the pandemic led to an acceleration of the digitization of lifestyles, especially social interactions, which exacerbated the digital divide in society [55]. Older adults in the APIC-RCT were no exception. The appropriation and use of technology were uneven among older adults and volunteers, despite the organizations’ support. Although some older adults were able to benefit from the APIC through virtual participation, a lack of skills or no access to technology hindered the participation of others. As described in the study by Poulin and colleagues [46], the pandemic also exacerbated the difficulty of recruiting volunteers. Because it involved both older adults and volunteers, the APIC-RCT was particularly affected by recruitment difficulties. Indeed, in this context, it was challenging to involve older adults and volunteers (often, older adults themselves) who had to discontinue attending face-to-face meetings in order to respect the guidelines and protect their health. The digital divide was also observed between the research team and community organizations. These organizations had less access to equipment and support for the appropriation and use of technology, especially during work from home, which often involved family constraints [56], a context that should also be considered when working in partnership.

### Implications for practice and future study

The importance of obtaining the appropriate resources to carry out this type of study is fundamental, especially in the context of a pandemic. This study was one of the rare projects to benefit from additional funding for the research because of this context. Moreover, a close partnership with community organizations was essential for the success of this pragmatic RCT in each phase of the study (including its conceptualization). Although low response and appreciable dropout rates had already been identified in other RCTs of social interventions [17–24], to ensure great scientific rigor it is important to be aware of the ethical and recruitment challenges in implementation in real-world settings. The research should be realistically and carefully planned to track progress and success as well as identify and address potential problems. Identifying problems and applying appropriate mitigation strategies facilitate quick feedback and revision of the plan, which is also facilitated by continuous and comprehensive monitoring, and the collaboration of partners and the research team (*e.g.*, supervisory committee). Moreover, finding additional funding was an important strategy for the APIC-RCT’s success. Financial support of pragmatic RCTs can be obtained through various networks, such as the pan-Canadian Strategy for Patient-Oriented Research (SPOR) support network [57] and the Quebec Network for Research on Aging [58].

Because it aims to empower isolated older adults by developing their social participation through pairing with volunteers and support from organizations, the APIC is an intervention that gives older adults’ new opportunities to increase community integration and enhance the social component of their lives [33]. Although complex, more research should be carried out on this social intervention which involves essential peer support, complements professional services [25] and is increasingly important as populations get older, more diverse and multicultural [25]. As aging education and intergenerational interventions have been reported to have a positive impact on people’s attitudes, knowledge and comfort with regard to older adults [59], the APIC provides an opportunity for younger volunteers to contribute to society. The APIC can also enhance older adults’ role in the community, reduce the consequences of ageism, present a realistic and nuanced vision of aging [60], and help create opportunities for a more inclusive society.

### Study strengths and limitations

To our knowledge, this multiple case study using the well-established CFIR is the first to have documented the strengths, challenges, and strategies of a pragmatic multicenter RCT which evaluates an intervention fostering older adults’ health and social participation. Triangulation of data from the research team and several partners in different organizational contexts provided original information on strategies to carry out an RCT in a real-world setting. However, social desirability may have inhibited some participants, even though they were informed of the importance of answering as accurately as possible and the absence of right or wrong answers. Since data were collected remotely to ensure no delay and the contribution of all participants geographically dispersed across Quebec, and in view of the measures associated with the COVID-19 pandemic, it is possible that some information was not fully captured or was misinterpreted (e.g., environmental distraction due to virtual participation), which may have impacted the results (e.g., less in-depth exploration). Like other qualitative studies, the findings are context- and time-sensitive (this RCT was conducted before, during, and after a historic pandemic). Also, similar to other qualitative studies, the results reflect the research team’s interpretation.

## Conclusions

This multiple case study explored the strengths, challenges, and strategies for improving the implementation of a pragmatic multicenter, prospective, two-arm RCT evaluating the effects of the Personalized Citizen Assistance for Social Participation, the APIC, on older adults’ health, social participation, and life satisfaction. Despite generating results with a high level of evidence, this pragmatic RCT presented a number of challenges associated with the implementation of an innovation in a real-world context. Although everyone believed in the strengths of the APIC-RCT, the design considerably limited organizations’ ability to recruit older adults and volunteers. The five participating nonprofit community organizations, the cooperative, and the research team found innovative strategies to mitigate the challenges encountered. Their collaboration was essential to the success of the APIC-RCT. The strategies identified should guide future RCT studies. More research is also needed to investigate the fidelity of the implementation of the APIC-RCT in these organizations.

### Supplementary Information

Supplementary material 1.

Supplementary material 2.

## Data Availability

Data are available from the corresponding author upon request.

## Abbreviations

APIC: Personalized Citizen Assistance for Social Participation
FRQS: *Fonds de la recherche du Québec* − *Santé*
CIHR: Canadian Institutes of Health Research
