# Supplementary material for: Strengths, challenges, and strategies for implementing pragmatic multicenter randomized controlled trials (RCTs): example of the Personalized Citizen Assistance for Social Participation (APIC) trial
Source: Trials. 2024 Jun 27;25:415. doi: 10.1186/s13063-024-08248-w (PMC11210038; doi:10.1186/s13063-024-08248-w)
Supplement: Supplementary file 1 — Supplementary material 1. [file 13063_2024_8248_MOESM1_ESM.pdf]

SPIRIT

STANDARD PROTOCOL ITEMS: RECOMMENDATIONS FOR INTERVENTIONAL TRIALS

SPIRIT 2013 Checklist: Recommended items to address in a clinical trial protocol and related documents

Section/item	Item No	Description
**Administrative information**		
**Title**	**1**	Strengths, challenges, and strategies for implementing pragmatic multicenter randomized controlled trials (RCTs): example of the Personalized Citizen Assistance for Social Participation (APIC) trial
**Trial registration**	**2a**	**Trial registration:** NCT03161860; Pre-results.
	**2b**	Please refer to item 2a.
**Protocol version**	**3**	The latest version of the protocol dates from October 6, 2020.
**Funding**	**4**	Funding was obtained from the Canadian Institutes of Health Research (CIHR; grant #284179). [First author] is a *Fonds de la recherche du Québec – Santé* (*FRQS*) Senior Researcher (#298996; 2021-2025) who now holds a Tier 1 Canadian Research Chair in Social Participation and Connection for Older Adults (CRC-2022-00331; 2023-2030).
**Roles and responsibilities**	**5a**	Mélanie Levasseur[1,2*], OT, PhD, Agathe Chaintré-Prieur[1,2], RN, MA, Marie-France Dubois[1,2], PhD, Catherine Maisonneuve[1], MSc, Johanne Filiatrault[3,4], OT, PhD, and Helen-Maria Vassiliadis[1], PhD<br><br>[1] Faculty of Medicine and Health Sciences, *Université de Sherbrooke*, Sherbrooke, Québec, Canada

[2] Research Centre on Aging, Eastern Townships Integrated University Health and Social Services Centre – Sherbrooke University Hospital Centre, Québec, Canada
[3] Research Centre, University Institute of Geriatrics of Montreal (CRIUGM), Québec, Canada
[4] Faculty of Medicine and Health Sciences, *Université de Montréal*, Montréal, Québec, Canada

5b **Canadian Institutes of Health Research**

160 Elgin Street, 10th Floor
Address Locator 4809A
Ottawa ON K1A 0W9
Canada

Telephone: 613-941-2672

5c The sponsor played no part in study design; collection, management, analysis, or interpretation of data; writing of the report; or the decision to submit the report for publication.

5d Composition of the steering commitee:

CO-RESEARCHERS:
Levasseur, Mélanie[1,2], OT, PhD (principal investigator)
Dubois, Marie- France[1,2], PhD
Filiatrault, Johanne[3,4], OT, PhD
Vasiliadis, Helen-Maria[5,6], PhD

METHODOLOGICAL EXPERTS
Lefebvre, Hélène[9,10], RN, PhD (Education)
Levert, Marie-Josée[9,10], Psy, PhD
Tourigny, André[7,8], MD, MBA (Public Health abd Preventive Medicine)
Gabaude, Catherine[11], PhD
Berger, Valérie[12], PhD
Eymard, Chantal[13], PhD (Nursing)

RESEARCH COORDINATORS
Lacasse-Bédard, Joanie[1], MA *(10/2016 to 07/2018 & 09/2020-09/2021 & 07/2022-07/2023)*

Maisonneuve, Catherine[2], MSc *(08/2018-08/2020 [maternity leave from 09/2020 to 09/2021] & 10/2021/06/2022)*
Prieur Chaintré, Agathe[1,2], MA *(08/2023-present)*

1. *Research Centre on Aging, Centre intégré universitaire de santé et de services sociaux (CIUSSS) de l'Estrie, Centre hospitalier universitaire de Sherbrooke (CHUS), 1036 Belvedere South, Sherbrooke, Québec, J1H 4C4, Canada*
2. *Faculty of Medicine and Health Sciences, Université de Sherbrooke, 3001 12th Avenue North, Sherbrooke, Québec, J1H 5N4, Canada*
3. *Research Centre, Institut universitaire de gériatrie de Montreal (CRIUGM), 4565 Queen Mary Road, Montréal, Québec, H3W 1W5, Canada*
4. *School of Rehabilitation, Université de Montréal, 7077 Park Avenue, Montréal, Québec, H3C 3J7, Canada*
5. *Research Centre, Charles-Le Moyne Hospital, Université de Sherbrooke Longueuil Campus, 150 Charles-LeMoyne Place, Suite 200, Longueuil, Québec, J4K 0A8, Canada*
6. *Department of Community Health Sciences, Université de Sherbrooke, 2500 University Boulevard, Sherbrooke, Québec, J1K 2R1, Canada*
7. *Department of Social and Preventive Medicine, Laval University, 1050 Medicine Avenue, Suite 4633, Québec City, Québec, G1V 0A6, Canada*
8. *Institute on Aging and Seniors' Social Participation, Suite L2-44, Saint-Sacrement Hospital, 1050 Sainte-Foy Street, Québec City, Québec, G1S 4L8, Canada*
9. *Centre for Interdisciplinary Research in Rehabilitation of Greater Montreal, 2275 Laurier Avenue East, Montréal, Québec, H2H 2N8, Canada*
10. *Faculty of Nursing, Université de Montréal, J.-A.-DeSève Building, 3rd Floor, 2332 Édouard-Montpetit Boulevard, Montréal, Québec, H3T 1J4, Canada*
11. *French Institute of Transport, Development and Network Science and Technology (IFSTTAR), 14-20 Newton Cité Descartes Boulevard, Champs sur Marne F-77447 Marne la Vallée Cedex 2, France*
12. *University Hospital of Bordeaux, 3 Ter Place de la Victoire, 33000 Bordeaux Cedex, France*

13. Aix-Marseille University, Jardin du Pharo, 58 Charles Livon Boulevard, 13284 Marseille, France

**Introduction**		
**Background and rationale**	**6a**	Pages 2-6
	**6b**	This study is a qualitative study carried out on the implementation of a pragmatic multicenter, prospective, two-arm RCT.
**Objectives**	**7**	Page 6
**Trial Design**	**8**	Pages 6-7
**Methods: Participants, interventions, and outcomes**		
**Study setting**	**9**	Pages 6-7
**Eligibility criteria**	**10**	Pages 7-8
**Interventions**	**11a**	NA. This study is a qualitative study carried out on the implementation of a pragmatic multicenter, prospective, two-arm RCT. The participants did not receive any interventions but implemented the intervention.
	**11b**	NA. This study is a qualitative study carried out on the implementation of a pragmatic multicenter, prospective, two-arm RCT. The participants did not receive any interventions but implemented the intervention.
	**11c**	NA. This study is a qualitative study carried out on the implementation of a pragmatic multicenter, prospective, two-arm RCT. The participants did not receive any interventions but implemented the intervention.
	**11d**	NA. This study is a qualitative study carried out on the implementation of a pragmatic multicenter, prospective, two-arm RCT. The participants did not receive any interventions but implemented the intervention.
**Outcomes**	**12**	NA. This study is a qualitative study carried out on the implementation of a pragmatic

		multicenter, prospective, two-arm RCT. The participants did not receive any interventions but implemented the intervention.
**Participant timelime**	**13**	NA. This study is a qualitative study carried out on the implementation of a pragmatic multicenter, prospective, two-arm RCT. The participants did not receive any interventions but implemented the intervention.
**Sample size**	**14**	NA. This study is a qualitative study carried out on the implementation of a pragmatic multicenter, prospective, two-arm RCT. The design is a multiple case study.
**Recruitment**	**15**	Participants were recruited from organizations implementing the intervention (APIC) as part of the APIC-RCT.

Methods: Assignment of interventions (for controlled trials)

Allocation:

**Sequence generation**	**16a**	NA. This study is a qualitative study carried out on the implementation of a pragmatic multicenter, prospective, two-arm RCT.
**Allocation concealment mecanism**	**16b**	NA. This study is a qualitative study carried out on the implementation of a pragmatic multicenter, prospective, two-arm RCT.
**Implementation**	**16c**	NA. This study is a qualitative study carried out on the implementation of a pragmatic multicenter, prospective, two-arm RCT.
**Blinding (masking)**	**17a**	NA. This study is a qualitative study carried out on the implementation of a pragmatic multicenter, prospective, two-arm RCT.
	**17b**	NA. This study is a qualitative study carried out on the implementation of a pragmatic multicenter, prospective, two-arm RCT.

Methods: Data collection, management, and analysis

**Data collection**	**18a**	Pages 8-9

	**18b**	NA. This study is a qualitative study carried out on the implementation of a pragmatic multicenter, prospective, two-arm RCT. The participants did not receive any interventions but implemented the intervention.
**Data management**	**19**	The qualitative data collected were stored on a secure S*harepoint*.
**Statistical methods**	**20a**	Page 10
	**20b**	Page 10
	**20c**	NA. This study is a qualitative study carried out on the implementation of a pragmatic multicenter, prospective, two-arm RCT. The participants did not receive any interventions but implemented the intervention.
**Methods: Monitoring**		
**Data monitoring**	**21a**	NA. This study is a qualitative study carried out on the implementation of a pragmatic multicenter, prospective, two-arm RCT.
	**21b**	There were no interim analyses for this study.
**Harms**	**22**	There were no adverse events for this study.
**Auditing**	**23**	NA. This study is a qualitative study carried out on the implementation of a pragmatic multicenter, prospective, two-arm RCT.
**Ethics and dissemination**		
**Research ethics and approval**	**24**	The study was approved by the Research Ethics Committee of the University Institute of Geriatrics of Sherbrooke Health and Social Services Centre (MP-22-2014-383).
**Protocol amendments**	**25**	There were no protocol amendments for this study.
**Consent or assent**	**26a**	A research coordinator from the principal investigator’s team was the person responsible for obtaining informed consent or assent from potential participants.

	**26b**	NA. This study is a qualitative study carried out on the implementation of a pragmatic multicenter, prospective, two-arm RCT.
**Confidentiality**	**27**	All data collected were anonymized when recorded in a secure file.
**Declaration of interests**	**28**	The authors declare that they have no competing interests.
**Access to data**	**29**	Data are available from the corresponding author (principal investigator) upon request.
**Ancillary and post-trial care**	**30**	NA. This study is a qualitative study carried out on the implementation of a pragmatic multicenter, prospective, two-arm RCT.
**Dissemination policy**	**31a**	The results of this study will be disseminated through scientific publications and presentations at national and international conferences.
	**31b**	The eligibility of authors meets the following 4 criteria:<br>1. Contribute significantly to at least one of the following 3 stages:<br>*-Design and implementation of the work plan*<br>*-Carrying out experiments or collecting data*<br>*-Analysis and interpretation of results*<br>2. Participate in the drafting or critical revision of the intellectual content of the document<br>3. Approve the final version of the document<br>4. Defend the outline of the document and the content corresponding to his/her contribution.<br>Finally, a professional English translator revised the language and grammar of the document.
	**31c**	Protocol and dataset are available from the corresponding author (principal investigator) upon request.
**Appendices**		
**Informed consent materials**	**32**	See Appendices

**Biological specimens**	**33**	NA. This study is a qualitative study carried out on the implementation of a pragmatic multicenter, prospective, two-arm RCT.

***It is strongly recommended that this checklist be read in conjunction with the SPIRIT 2013 Explanation & Elaboration for important clarification on the items. Amendments to the protocol should be tracked and dated. The SPIRIT checklist is copyrighted by the SPIRIT Group under the Creative Commons "Attribution-NonCommercial-NoDerivs 3.0 Unported" license.**

FORMULAIRE D'INFORMATION ET DE CONSENTEMENT À LA RECHERCHE (COORDONNATEUR)

Titre du projet : Documentation de l'implantation du programme d'Accompagnement-citoyen personnalisé d'intégration communautaire (APIC) dans 5 organismes communautaires du Québec

Numéro du projet : MP-31-2018-2424

Organisme subventionnaire Institut de recherche en santé du Canada (IRSC)

Équipe de recherche

Chercheure responsable
Mélanie Levasseur, erg., Ph.D., CdRV du CIUSSS de l'Estrie-CHUS

Agente de recherche
Joanie Lacasse-Bédard, M.A., CdRV du CIUSSS de l'Estrie-CHUS

Étudiantes-chercheures (étudiantes à la maîtrise en ergothérapie)
Anne-Marie Duguay
Véronique Gaumond
Stéphanie Germain
Audrey Vézina

POUR INFORMATION

Du lundi au vendredi entre 8 h 30 et 16 h 30, vous pouvez communiquer avec :

Joanie Lacasse-Bédard, Agente de recherche — Tél : (819) 780-2220, poste 45415

Mélanie Levasseur, erg., Ph.D. Chercheure responsable — Tél : (819) 821-8000, poste 72927

Nous sollicitons votre participation à un projet de recherche parce qu'en tant que coordonnateur, vous êtes un acteur clé quant à l'implantation de l'APIC dans votre communauté et une source d'information de choix.

Cependant, avant d'accepter de participer à ce projet, veuillez prendre le temps de lire, de comprendre et de considérer attentivement les renseignements qui suivent. Si vous acceptez de participer au projet de recherche, vous devrez signer le formulaire de consentement à la fin du présent document et nous vous en remettrons une copie pour vos dossiers.

Ce formulaire d'information et de consentement vous explique le but de ce projet de recherche, les procédures, les risques et inconvénients ainsi que les avantages, de même que les personnes avec qui communiquer au besoin. Il peut contenir des mots que vous ne comprenez pas. Nous vous invitons à poser toutes les questions nécessaires au chercheur responsable du projet ou aux autres personnes affectées au projet de recherche et à leur demander de vous expliquer tout mot ou renseignement qui n'est pas clair.

NATURE ET OBJECTIFS DU PROJET DE RECHERCHE

Actuellement, les interventions visant à favoriser la participation sociale des personnes âgées vivant à domicile demeurent rares et discontinues au Québec. De plus, on connaît peu les facteurs favorables et défavorables à l'implantation d'intervention visant à favoriser la participation sociale des aînés, d'où la pertinence de ce projet de recherche, qui a pour objectif de documenter l'implantation d'une intervention d'Accompagnement-citoyen personnalisé d'intégration communautaire (APIC) dans 5 organismes communautaires du Québec offrant des activités et services aux aînés présentant des incapacités.

DÉROULEMENT DU PROJET DE RECHERCHE

Votre participation à cette recherche consiste à être observé lors de deux rencontres mensuelles avec les accompagnateurs-bénévoles de votre milieu, à accorder deux entretiens individuels semi-dirigés, ainsi qu'à compléter un questionnaire sociodémographique.

<u>Observation de deux rencontres mensuelles avec les accompagnateurs-bénévoles</u>

Vous serez observé lors de votre animation de deux rencontres mensuelles avec les accompagnateurs-bénévoles de votre milieu afin de document l'implantation dans votre communauté. Ces deux rencontres seront sélectionnées au hasard (environ à 6 et 12 mois suivant le début de l'implantation).

<u>Entretiens semi-dirigés</u>

À la suite de l'observation de chacune de ces rencontres mensuelles, vous devrez accorder un entretien individuel visant à documenter l'implantation de l'APIC dans votre communauté. Ces rencontres, réalisées dans un lieu à votre convenance, seront d'une durée de 60 à 90 minutes. Au besoin, un entretien

supplémentaire pourrait avoir lieu afin de clarifier et approfondir certains thèmes abordés.

Un compte-rendu de chacun des entretiens vous sera envoyé par courriel et un membre de l'équipe de recherche communiquera avec vous dans la semaine suivant la rencontrer afin de valider ces informations.

Chaque entretien sera enregistré sur une bande audionumérique.

Questionnaire sociodémographique

Nous vous demanderons également de compléter un questionnaire sur vos caractéristiques personnelles lors du premier entretien.

INCONVÉNIENTS POUVANT DÉCOULER DE VOTRE PARTICIPATION AU PROJET DE RECHERCHE

Il n'y a pas d'inconvénients à prendre part à l'étude, hormis le temps nécessaire pour les entretiens et compléter le questionnaire sociodémographique. Il vous sera en tout temps possible de prendre une pause, si vous le désirez.

AVANTAGES POUVANT DÉCOULER DE VOTRE PARTICIPATION AU PROJET DE RECHERCHE

Il se peut que vous retiriez un bénéfice personnel de votre participation à ce projet de recherche, mais nous ne pouvons vous l'assurer. Par ailleurs, les informations découlant de ce projet de recherche pourraient contribuer à l'avancement des connaissances relatives aux interventions de participation sociale offertes aux aînés.

PARTICIPATION VOLONTAIRE ET POSSIBILITÉ DE RETRAIT

Votre participation à ce projet de recherche est volontaire. Vous êtes donc libre de refuser d'y participer. Vous pouvez également vous retirer de ce projet à n'importe quel moment, sans avoir à donner de raison, en informant l'équipe de recherche. Votre décision de ne pas participer à ce projet de recherche ou de vous en retirer n'aura aucune conséquence sur votre relation avec les équipes qui les dispensent.

Le responsable de ce projet de recherche, le comité d'éthique de la recherche ou l'organisme subventionnaire peuvent mettre fin à votre participation, sans votre consentement. Cela peut se produire si de nouvelles découvertes ou informations indiquent que votre participation au projet n'est plus dans votre intérêt, si vous ne respectez pas les consignes du projet de recherche ou encore s'il existe des raisons administratives d'abandonner le projet.

Si vous vous retirez du projet ou êtes retiré du projet, les informations déjà recueillies seront néanmoins conservées, analysées ou utilisées pour assurer l'intégrité du projet.

Toute nouvelle connaissance acquise durant le déroulement du projet qui pourrait avoir un impact sur votre décision de continuer à participer à ce projet vous sera communiquée rapidement.

CONFIDENTIALITÉ

Durant votre participation à ce projet de recherche, les membres de l'équipe de recherche recueilleront, dans un dossier de recherche, les renseignements vous concernant et nécessaires pour répondre aux objectifs de ce projet de recherche.

Ces renseignements comprendront les comptes rendus des entretiens ainsi que des renseignements sociodémographiques tels que votre âge et votre scolarité auxquels vous aurez répondu durant ce projet.

Tous ces renseignements demeureront strictement confidentiels dans les limites prévues par la loi. Afin de préserver votre identité et la confidentialité des renseignements, vous ne serez identifié(e) que par un numéro de code. La clé du code reliant votre nom à votre dossier de recherche sera conservée par les chercheurs responsables pour une durée de 5 ans suivant la fin du projet. Cette clé sera ensuite détruite, de manière sécuritaire, selon les normes en vigueur au Comité d'éthique de la recherche du CIUSSS de l'Estrie-CHUS.

Les résultats recueillis dans le cadre de ce projet de recherche pourront être publiés dans des revues spécialisées ou faire l'objet de discussions scientifiques. Cependant, aucune publication ou communication scientifique ne renfermera quoi que ce soit qui puisse permettre de vous identifier.

Concernant les bandes audionumériques, ces dernières seront détruites aussitôt les comptes rendus retranscrits.

À des fins de surveillance et de contrôle, votre dossier de recherche pourra être consulté par des personnes mandatées par le Comité d'éthique de la recherche du CIUSSS de l'Estrie-CHUS, par l'établissement ou par une personne nommée par un organisme autorisé. Toutes ces personnes et ces organismes adhèrent à une politique de stricte confidentialité.

Vous avez le droit de consulter votre dossier de recherche pour vérifier les renseignements recueillis et les faire rectifier au besoin, et ce, aussi longtemps que les responsables du projet détiennent ces informations. Cependant, afin de préserver l'intégrité scientifique du projet, vous pourriez n'avoir accès qu'à certaines de ces informations qu'une fois votre participation terminée.

COMPENSATION

Vous ne recevrez pas de compensation financière pour votre participation à ce projet de recherche.

EN CAS DE PRÉJUDICE

En acceptant de participer à ce projet de recherche, vous ne renoncez à aucun de vos droits et vous ne libérez pas les responsables de ce projet de recherche,

l'organisme subventionnaire et l'établissement de leur responsabilité civile et professionnelle.

PERSONNES-RESSOURCES
Si vous avez des questions ou éprouvez des problèmes en lien avec le projet de recherche ou si vous souhaitez vous en retirer, vous pouvez communiquer avec une personne de l'équipe de recherche aux numéros suivants :

- Joanie Lacasse-Bédard, M.A., agente de recherche : 1 888 780-8990 (sans frais)
- Mélanie Levasseur, erg., Ph.D. : 819 821-8000, poste 72927

SURVEILLANCE DES ASPECTS ÉTHIQUES
Le Comité d'éthique de la recherche du CIUSSS de l'Estrie-CHUS a approuvé le projet et en assurera le suivi du projet. Si vous désirez rejoindre l'un des membres de ce comité, vous pouvez communiquer avec le Service de soutien à l'éthique de la recherche du CIUSSS de l'Estrie - CHUS au numéro 819-346-1110, poste 12856.

Pour toute question concernant vos droits en tant que participant à ce projet de recherche ou si vous avez des plaintes à formuler, vous pouvez également communiquer avec le Service de soutien à l'éthique de la recherche du CIUSSS de l'Estrie-CHUS au numéro 819-346-1110 poste 12856.

CONSENTEMENT

J'ai pris connaissance du présent formulaire d'information et de consentement. Je reconnais qu'on m'a expliqué le projet de recherche, qu'on a répondu à mes questions et qu'on m'a laissé le temps voulu pour prendre une décision.

Après réflexion, je consens à participer à ce projet de recherche aux conditions qui y sont énoncées. Une copie signée et datée du présent formulaire d'information et de consentement me sera remise.

Nom du participant *(lettres moulées)*	Signature du participant	Date

Je déclare avoir expliqué au participant les termes du projet de recherche et du présent formulaire d'information et de consentement et répondu aux questions qu'il m'a posées. Une copie signée et datée du document lui a été remise.

Nom de la personne qui obtient le consentement *(lettres moulées)*	Signature	Date

ENGAGEMENT DU CHERCHEUR

Je certifie qu'on a expliqué au participant les termes du présent formulaire d'information et de consentement, que l'on a répondu aux questions qu'il avait à cet égard et qu'on lui a clairement indiqué qu'il demeure libre de mettre un terme à sa participation, et ce, sans préjudice.

Je m'engage, avec l'équipe de recherche, à respecter ce qui a été convenu au formulaire d'information et de consentement et à en remettre une copie signée et datée au participant.

Nom du chercheur *(lettres moulées)*	Signature	Date

Subscribe to DeepL Pro to translate larger documents.
Visit www.DeepL.com/pro for more information.

RESEARCH INFORMATION AND CONSENT FORM (COORDINATOR)

Project title : Program implementation documentation of Accompagnement-citoyen personnalisé d'intégration communautaire (APIC) in 5 Quebec community organizations

Project number: MP-31-2018-2424

Organization grantor Canadian Institutes of Health Research (CIHR)

Research team

Researcher in charge
Mélanie Levasseur, erg., Ph.D., CdRV of CIUSSS de l'Estrie-CHUS

Research Officer
Joanie Lacasse-Bédard, M.A., CdRV du CIUSSS de l'Estrie-CHUS

Student researchers (master's students in occupational therapy)
Anne-Marie Duguay
Véronique Gaumond
Stéphanie Germain
Audrey Vézina

FOR INFORMATION

From Monday to Friday between 8:30 a.m. and 4:30 p.m., you can contact :

Joanie Lacasse-Bédard, Research Officer — Tel: (819) 780-2220, ext. 45415

Mélanie Levasseur, erg., Ph.D. Responsible researcher — Tel: (819) 821-8000, ext. 72927

We're asking you to take part in a research project because, as a coordinator, you're a key player in the implementation of APIC in your community, and a prime source of information.

However, before agreeing to participate in this project, please take the time to read, understand and carefully consider the following information. If you agree to participate in this research project, you will be asked to sign the consent form at the end of this document, and we will provide you with a copy for your records.

This information and consent form explains the purpose of this research project, the procedures, the risks and inconveniences as well as the benefits, and who to contact if necessary. It may contain words you do not understand. We invite you to ask any questions you may have to the researcher in charge of the project or other people involved in the research project, and to ask them to explain any words or information that are not clear.

NATURE AND OBJECTIVES OF THE RESEARCH PROJECT

Currently, interventions aimed at promoting the social participation of seniors living at home remain rare and discontinuous in Quebec. Moreover, little is known about the factors favoring and hindering the implementation of interventions designed to promote the social participation of seniors, hence the relevance of this research project, which aims to document the implementation of an Accompagnement-citoyen personnalisé d'intégration communautaire (APIC) intervention in 5 Quebec community organizations offering activities and services to seniors with disabilities.

PROGRESS OF THE RESEARCH PROJECT

Your participation in this research consists of being observed during two monthly meetings with volunteer coaches in your area, giving two semi-structured individual interviews, and completing a socio-demographic questionnaire.

<u>Observation of two monthly meetings with volunteer coaches</u>

During your facilitation, you will be observed at two monthly meetings with the volunteer coaches in your area, in order to document the implementation in your community. These two meetings will be selected at random (approximately 6 and 12 months after the start of implementation).

<u>Semi-structured interviews</u>

Following the observation of each of these monthly meetings, you will be asked to conduct a one-on-one interview to document the implementation of APIC in your community. These meetings, held at a location of your choice, will last between 60 and 90 minutes. If necessary, a

could be held to clarify and deepen certain topics.

A report of each interview will be sent to you by e-mail, and a member of the research team will contact you within a week of the meeting to validate this information.

Each interview will be recorded on digital audio tape.

Socio-demographic questionnaire

We'll also ask you to complete a questionnaire about your personal characteristics during the first interview.

DISADVANTAGES THAT MAY ARISE FROM YOUR PARTICIPATION IN THE RESEARCH PROJECT

There are no disadvantages to taking part in the study, apart from the time required for interviews and completing the socio-demographic questionnaire. You can take a break at any time, if you wish.

ADVANTAGES CAN BECOME FROM YOUR PARTICIPATION IN THE RESEARCH PROJECT

You may derive some personal benefit from your participation in this research project, but we cannot assure you of this. On the other hand, the information derived from this research project could contribute to the advancement of knowledge about social participation interventions for seniors.

VOLUNTARY PARTICIPATION AND OPT-OUT

Your participation in this research project is voluntary. You are therefore free to refuse to take part. You may also withdraw from this project at any time, without giving any reason, by informing the research team. Your decision not to participate or to withdraw from the research project will have no effect on your relationship with the research team.

The person in charge of this research project, the research ethics committee or the funding organization may terminate your participation without your consent. This can happen if new findings or information indicate that your participation in the project is no longer in your best interest, if you fail to comply with the research project guidelines, or if there are administrative reasons for abandoning the project.

If you withdraw or are withdrawn from the project, the information already collected will nevertheless be retained, analyzed or used to ensure the integrity of the project.

Any new knowledge acquired during the course of the project that could have an impact on your decision to continue participating in this project will be communicated to you promptly.

PRIVACY

During your participation in this research project, members of the research team will collect, in a research file, information about you that is necessary to meet the objectives of this research project.

This information will include interview reports as well as socio-demographic information such as your age and education, which you will have answered during this project.

All such information will remain strictly confidential to the extent permitted by law. To preserve your identity and the confidentiality of the information, you will be identified only by a code number. The key to the code linking your name to your research file will be kept by the researchers in charge for a period of 5 years following the end of the project. This key will then be securely destroyed in accordance with the standards in force at the CIUSSS de l'Estrie-CHUS Research Ethics Board.

The results of this research project may be published in specialized journals or discussed in scientific circles. However, no publication or scientific communication will contain anything that could identify you.

Digital audio tapes will be destroyed as soon as the reports have been transcribed.

For monitoring and control purposes, your research file may be consulted by persons mandated by the CIUSSS de l'Estrie-CHUS Research Ethics Board, by the establishment or by a person appointed by an authorized organization. All these persons and organizations adhere to a policy of strict confidentiality.

You have the right to consult your research file to verify the information collected and have it corrected if necessary, for as long as the people in charge of the project hold this information. However, in order to preserve the scientific integrity of the project, you may only have access to some of this information once your participation has ended.

COMPENSATION

You will not receive any financial compensation for your participation in this research project.

IN THE EVENT OF LOSS

By agreeing to participate in this research project, you do not waive any of your rights and you do not release those responsible for this research project,

and the establishment of their civil and professional liability.

CONTACTS
If you have any questions or problems in connection with the research project, or if you wish to withdraw, you can contact a member of the research team at the following numbers:

- Joanie Lacasse-Bédard, M.A., Research Officer: 1 888 780-8990 (toll-free)
- Mélanie Levasseur, erg., Ph.D.: 819 821-8000, ext. 72927

MONITORING ETHICAL ASPECTS
The CIUSSS de l'Estrie-CHUS Research Ethics Committee has approved the project and will monitor its progress. If you would like to contact a member of this committee, please contact the CIUSSS de l'Estrie - CHUS Research Ethics Support Department at 819-346-1110, ext. 12856.

If you have any questions about your rights as a participant in this research project, or if you have any complaints, you can also contact the CIUSSS de l'Estrie-CHUS Research Ethics Support Department at 819-346-1110, ext. 12856.

CONSENT

I have read this information and consent form. I acknowledge that the research project has been explained to me, that my questions have been answered and that I have been given sufficient time to make a decision.

After consideration, I consent to participate in this research project under the conditions stated herein. A signed and dated copy of this information and consent form will be given to me.

Name of participant *(block letters)*	Signature of participant	Date

I declare that I have explained to the participant the terms of the research project and of this information and consent form, and that I have answered the questions he asked me. A signed and dated copy of the document has been given to the participant.

Name of person who consent obtained *(block letters)*	Signature	Date

THE RESEARCHER'S COMMITMENT

I certify that the terms of this information and consent form have been explained to the participant, that any questions he or she may have in this regard have been answered, and that it has been made clear to him or her that he or she remains free to terminate his or her participation without prejudice.

I undertake, together with the research team, to respect what has been agreed in the information and consent form and to give a signed and dated copy to the participant.

Name of researcher *(block letters)*	Signature	Date

FORMULAIRE D'INFORMATION ET DE CONSENTEMENT A LA RECHERCHE (AGENTE DE RECHERCHE)

Titre du projet : Documentation de l'implantation du programme d'Accompagnement-citoyen personnalisé d'intégration communautaire (APIC) dans 5 organismes communautaires du Québec

Numéro du projet : MP-31-2018-2424

Organisme subventionnaire Institut de recherche en santé du Canada (IRSC)

Équipe de recherche

Chercheure responsable
Mélanie Levasseur, erg., Ph.D., CdRV du CIUSSS de l'Estrie-CHUS

Étudiantes-chercheures (étudiantes à la maîtrise en ergothérapie)
Anne-Marie Duguay
Véronique Gaumond
Stéphanie Germain
Audrey Vézina

POUR INFORMATION

Du lundi au vendredi entre 8 h 30 et 16 h 30, vous pouvez communiquer avec :

Mélanie Levasseur, erg., Ph.. Tél. : (819) 821-8000, poste 72927
Chercheure responsable

Nous sollicitons votre participation à un projet de recherche parce qu'en tant qu'agente de recherche, vous êtes une source d'information clé de l'implantation de l'APIC dans les communautés de par votre rôle qui vous amène à travailler en étroite collaboration avec les coordonnateurs des organismes communautaires impliqués.

Cependant, avant d'accepter de participer à ce projet, veuillez prendre le temps de lire, de comprendre et de considérer attentivement les renseignements qui suivent. Si vous acceptez de participer au projet de recherche, vous devrez

signer le formulaire de consentement à la fin du présent document et nous vous en remettrons une copie pour vos dossiers.

Ce formulaire d'information et de consentement vous explique le but de ce projet de recherche, les procédures, les risques et inconvénients ainsi que les avantages, de même que les personnes avec qui communiquer au besoin. Il peut contenir des mots que vous ne comprenez pas. Nous vous invitons à poser toutes les questions nécessaires au chercheur responsable du projet ou aux autres personnes affectées à l'étude et à leur demander de vous expliquer tout mot ou renseignement qui n'est pas clair.

NATURE ET OBJECTIFS DU PROJET DE RECHERCHE

Actuellement, les interventions visant à favoriser la participation sociale des personnes âgées vivant à domicile demeurent rares et discontinues au Québec. De plus, on connaît peu les facteurs favorables et défavorables à l'implantation d'intervention visant à favoriser la participation sociale des aînés, d'où la pertinence de ce projet de recherche, qui a pour objectif de documenter l'implantation d'un d'Accompagnement-citoyen personnalisé d'intégration communautaire (APIC) dans 5 organismes communautaires du Québec offrant des activités et services aux aînés présentant des incapacités.

DÉROULEMENT DU PROJET DE RECHERCHE

Entretiens individuels

Votre participation à cette recherche consiste à accorder deux entretiens individuels semi-dirigés, environ à 6 et 12 mois suivant le début de l'implantation. Chaque entretien, réalisé dans un lieu à votre convenance, sera d'une durée de 60 à 90 minutes. Au besoin, un entretien supplémentaire pourrait être réalisé afin de clarifier et approfondir certains thèmes abordés.

Un compte-rendu de chacun des entretiens vous sera envoyé par courriel et un membre de l'équipe de recherche communiquera avec vous dans la semaine suivant la rencontre afin de valider ces informations.

Chaque entretien sera enregistré sur une bande audionumérique.

Questionnaire sociodémographique

Nous vous demanderons également de compléter un questionnaire sur vos caractéristiques personnelles lors du premier entretien.

INCONVÉNIENTS POUVANT DÉCOULER DE VOTRE PARTICIPATION AU PROJET DE RECHERCHE

Il n'y a pas d'inconvénients à prendre part à l'étude, hormis le temps nécessaire pour les entretiens et compléter le questionnaire sociodémographique. Il vous sera en tout temps possible de prendre une pause, si vous le désirez.

AVANTAGES POUVANT DÉCOULER DE VOTRE PARTICIPATION AU PROJET DE RECHERCHE

Il se peut que vous retiriez un bénéfice personnel de votre participation à ce projet de recherche, mais nous ne pouvons vous l'assurer. Par ailleurs, les informations découlant de ce projet de recherche pourraient contribuer à l'avancement des connaissances relatives aux interventions de participation sociale offertes aux aînés.

PARTICIPATION VOLONTAIRE ET POSSIBILITÉ DE RETRAIT

Votre participation à ce projet de recherche est volontaire. Vous êtes donc libre de refuser d'y participer. Vous pouvez également vous retirer de ce projet à n'importe quel moment, sans avoir à donner de raisons, en informant l'équipe de recherche. Votre décision de ne pas participer à ce projet de recherche ou de vous en retirer n'aura aucune conséquence sur votre relation avec les équipes qui les dispensent.

Le responsable de ce projet de recherche, le comité d'éthique de la recherche, l'organisme subventionnaire ou le commanditaire peuvent mettre fin à votre participation, sans votre consentement. Cela peut se produire si de nouvelles découvertes ou informations indiquent que votre participation au projet n'est plus dans votre intérêt, si vous ne respectez pas les consignes du projet de recherche ou encore s'il existe des raisons administratives d'abandonner le projet.

Si vous vous retirez du projet ou êtes retiré du projet, les informations déjà recueillies seront néanmoins conservées, analysées ou utilisées pour assurer l'intégrité du projet.

Toute nouvelle connaissance acquise durant le déroulement du projet qui pourrait avoir un impact sur votre décision de continuer à participer à ce projet vous sera communiquée rapidement.

CONFIDENTIALITÉ

Durant votre participation à ce projet de recherche, les membres de l'équipe de recherche recueilleront, dans un dossier de recherche, les renseignements vous concernant et nécessaires pour répondre aux objectifs de ce projet de recherche.

Ces renseignements comprendront le compte rendu de l'entretien ainsi que des renseignements sociodémographiques tels que votre âge et votre scolarité auxquels vous aurez répondu durant ce projet.

Tous ces renseignements demeureront strictement confidentiels dans les limites prévues par la loi. Afin de préserver votre identité et la confidentialité des renseignements, vous ne serez identifié(e) que par un numéro de code. La clé du code reliant votre nom à votre dossier de recherche sera conservée par les chercheurs responsables pour une durée de 5 ans suivant la fin du projet. Cette clé sera ensuite détruite, de manière sécuritaire, selon les normes en vigueur au Comité d'éthique de la recherche du CIUSSS de l'Estrie-CHUS.

Les résultats recueillis dans le cadre de ce projet de recherche pourront être publiés dans des revues spécialisées ou faire l'objet de discussions scientifiques.

Cependant, aucune publication ou communication scientifique ne renfermera quoi que ce soit qui puisse permettre de vous identifier.

Concernant les bandes audionumériques, ces dernières seront détruites aussitôt les comptes rendus retranscrits.

À des fins de surveillance et de contrôle, votre dossier de recherche pourra être consulté par des personnes mandatées par le Comité d'éthique de la recherche du CIUSSS de l'Estrie-CHUS, par l'établissement ou par une personne nommée par un organisme autorisé. Toutes ces personnes et ces organismes adhèrent à une politique de stricte confidentialité.

Vous avez le droit de consulter votre dossier de recherche pour vérifier les renseignements recueillis et les faire rectifier au besoin, et ce, aussi longtemps que les responsables du projet détiennent ces informations. Cependant, afin de préserver l'intégrité scientifique du projet, vous pourriez n'avoir accès qu'à certaines de ces informations qu'une fois votre participation terminée.

COMPENSATION

Vous ne recevrez pas de compensation financière pour votre participation à ce projet de recherche.

EN CAS DE PRÉJUDICE

En acceptant de participer à ce projet de recherche, vous ne renoncez à aucun de vos droits et vous ne libérez pas les responsables de ce projet de recherche, l'organisme subventionnaire et l'établissement de leur responsabilité civile et professionnelle.

PERSONNE-RESSOURCE

Si vous avez des questions ou éprouvez des problèmes en lien avec le projet de recherche ou si vous souhaitez vous en retirer, vous pouvez communiquer avec une personne de l'équipe de recherche au numéro suivant :

- Mélanie Levasseur, erg., Ph.D. : 819 821-8000, poste 72927

SURVEILLANCE DES ASPECTS ÉTHIQUES

Le Comité d'éthique de la recherche du CIUSSS de l'Estrie-CHUS a approuvé ce projet de recherche et en assurera le suivi.

Si vous désirez rejoindre l'un des membres de ce comité vous pouvez communiquer avec le Service de soutien à l'éthique de la recherche du CIUSSS de l'Estrie - CHUS au numéro 819-346-1110, poste 12856.

Pour toute question concernant vos droits en tant que participant à ce projet de recherche ou si vous avez des plaintes à formuler, vous pouvez communiquer avec le Service de soutien à l'éthique de la recherche du CIUSSS de l'Estrie – CHUS au numéro 819-346-1110 poste 12856.

CONSENTEMENT

J'ai pris connaissance du présent formulaire d'information et de consentement. Je reconnais qu'on m'a expliqué le projet de recherche, qu'on a répondu à mes questions et qu'on m'a laissé le temps voulu pour prendre une décision.

Après réflexion, je consens à participer à ce projet de recherche aux conditions qui y sont énoncées. Une copie signée et datée du présent formulaire d'information et de consentement me sera remise.

Nom du participant *(lettres moulées)*	Signature du participant	Date

Je déclare avoir expliqué au participant les termes du projet de recherche et du présent formulaire d'information et de consentement et répondu aux questions qu'il m'a posées. Une copie signée et datée du document lui a été remise.

Nom de la personne qui obtient le consentement *(lettres moulées)*	Signature	Date

ENGAGEMENT DU CHERCHEUR

Je certifie qu'on a expliqué au participant les termes du présent formulaire d'information et de consentement, que l'on a répondu aux questions qu'il avait à cet égard et qu'on lui a clairement indiqué qu'il demeure libre de mettre un terme à sa participation, et ce, sans préjudice.

Je m'engage, avec l'équipe de recherche, à respecter ce qui a été convenu au présent formulaire d'information et de consentement et à en remettre une copie signée et datée au participant.

Nom du chercheur (lettres moulées)	Signature	Date

Subscribe to DeepL Pro to translate larger documents.
Visit www.DeepL.com/pro for more information.

RESEARCH INFORMATION AND CONSENT FORM (RESEARCH OFFICER)

Project title : Program implementation documentation of Accompagnement-citoyen personnalisé d'intégration communautaire (APIC) in 5 Quebec community organizations

Project number: MP-31-2018-2424

Organization grantor Canadian Institutes of Health Research (CIHR)

Research team

Researcher in charge
Mélanie Levasseur, erg., Ph.D., CdRV of CIUSSS de l'Estrie-CHUS

Student researchers (master's students in occupational therapy)
Anne-Marie Duguay
Véronique Gaumond
Stéphanie Germain
Audrey Vézina

FOR INFORMATION

From Monday to Friday between 8:30 a.m. and 4:30 p.m., you can contact :

Mélanie Levasseur, erg., Ph. Tel: (819) 821-8000, ext. 72927 Researcher in charge

We're asking you to take part in a research project because, as a research officer, you're a key source of information for the implementation of APIC in the communities, as your role requires you to work closely with the coordinators of the community organizations involved.

However, before agreeing to participate in this project, please take the time to read, understand and carefully consider the following information. If you agree to participate in this research project, you will be required to

sign the consent form at the end of this document and we will provide you with a copy for your records.

This information and consent form explains the purpose of this research project, the procedures, the risks and inconveniences as well as the benefits, and who to contact if necessary. It may contain words you do not understand. We encourage you to ask any questions you may have to the investigator in charge of the project or other people involved in the study, and to ask them to explain any words or information that are not clear.

NATURE AND OBJECTIVES OF THE RESEARCH PROJECT

Currently, interventions aimed at promoting the social participation of seniors living at home remain rare and discontinuous in Quebec. Moreover, little is known about the factors favoring and hindering the implementation of interventions designed to promote the social participation of seniors. Hence the relevance of this research project, which aims to document the implementation of an Accompagnement-citoyen personnalisé d'intégration communautaire (APIC) in 5 Quebec community organizations offering activities and services to seniors with disabilities.

PROGRESS OF THE RESEARCH PROJECT

Individual interviews

Your participation in this research involves two individual semi-structured interviews, approximately 6 and 12 months after the start of implantation. Each interview will last between 60 and 90 minutes, and will take place at a location of your choice. If necessary, an additional interview may be held to clarify and deepen certain issues.

A report of each interview will be sent to you by e-mail, and a member of the research team will contact you within a week of the meeting to validate this information.

Each interview will be recorded on digital audio tape. Socio-

demographic questionnaire

We'll also ask you to complete a questionnaire about your personal characteristics during the first interview.

DISADVANTAGES THAT MAY ARISE FROM YOUR PARTICIPATION IN THE RESEARCH PROJECT

There are no disadvantages to taking part in the study, apart from the time required for interviews and completing the socio-demographic questionnaire. You can take a break at any time, if you wish.

BENEFITS OF PARTICIPATING IN THE RESEARCH PROJECT

You may derive some personal benefit from your participation in this research project, but we cannot assure you of this. On the other hand, the information derived from this research project could contribute to the advancement of knowledge about social participation interventions for seniors.

VOLUNTARY PARTICIPATION AND OPT-OUT

Your participation in this research project is voluntary. You are therefore free to refuse to take part. You may also withdraw from this project at any time, without giving reasons, by informing the research team. Your decision not to participate or to withdraw from the research project will have no effect on your relationship with the research team.

The person in charge of this research project, the research ethics committee, the funding agency or the sponsor may terminate your participation without your consent. This can happen if new findings or information indicate that your participation in the project is no longer in your best interest, if you fail to comply with the research project guidelines, or if there are administrative reasons to abandon the project.

If you withdraw or are withdrawn from the project, the information already collected will nevertheless be retained, analyzed or used to ensure the integrity of the project.

Any new knowledge acquired during the course of the project that could have an impact on your decision to continue participating in this project will be communicated to you promptly.

PRIVACY

During your participation in this research project, members of the research team will collect, in a research file, information about you that is necessary to meet the objectives of this research project.

This information will include the minutes of the interview as well as socio-demographic information such as your age and education, which you will have answered during this project.

All such information will remain strictly confidential to the extent permitted by law. To preserve your identity and the confidentiality of the information, you will be identified only by a code number. The key to the code linking your name to your research file will be kept by the researchers in charge for a period of 5 years following the end of the project. This key will then be securely destroyed in accordance with the standards in force at the CIUSSS de l'Estrie-CHUS Research Ethics Board.

The results of this research project may be published in specialized journals or be the subject of scientific discussions.

However, no publication or scientific communication will contain anything that could identify you.

Digital audio tapes will be destroyed as soon as the reports have been transcribed.

For monitoring and control purposes, your research file may be consulted by persons mandated by the CIUSSS de l'Estrie-CHUS Research Ethics Board, by the establishment or by a person appointed by an authorized organization. All these persons and organizations adhere to a policy of strict confidentiality.

You have the right to consult your research file to verify the information collected and have it corrected if necessary, for as long as the people in charge of the project hold this information. However, in order to preserve the scientific integrity of the project, you may only have access to some of this information once your participation has ended.

COMPENSATION
You will not receive any financial compensation for your participation in this research project.

IN THE EVENT OF LOSS
By agreeing to participate in this research project, you do not waive any of your rights, and you do not release the people in charge of this research project, the granting agency and the institution from their civil and professional liability.

CONTACT
If you have any questions or problems in connection with the research project, or if you wish to withdraw, you can contact a member of the research team at the following number:

- Mélanie Levasseur, erg., Ph.D.: 819 821-8000, ext. 72927

MONITORING ETHICAL ASPECTS
The CIUSSS de l'Estrie-CHUS Research Ethics Committee has approved this research project and will ensure its follow-up.

If you would like to join one of the members of this committee, please contact the CIUSSS de l'Estrie - CHUS Research Ethics Support Department at 819-346-1110, ext. 12856.

If you have any questions about your rights as a participant in this research project, or if you have any complaints, you can contact the CIUSSS de l'Estrie - CHUS Research Ethics Support Department at 819-346-1110, ext. 12856.

CONSENT

I have read this information and consent form. I acknowledge that the research project has been explained to me, that my questions have been answered and that I have been given sufficient time to make a decision.

After consideration, I consent to participate in this research project under the conditions stated herein. A signed and dated copy of this information and consent form will be given to me.

Name of participant *(block letters)*	Signature of participant	Date

I declare that I have explained to the participant the terms of the research project and of this information and consent form, and that I have answered the questions he asked me. A signed and dated copy of the document has been given to the participant.

Name of person who consent obtained *(block letters)*	Signature	Date

THE RESEARCHER'S COMMITMENT

I certify that the terms of this information and consent form have been explained to the participant, that any questions he or she may have in this regard have been answered, and that it has been made clear to him or her that he or she remains free to terminate his or her participation without prejudice.

I agree with the research team to abide by the terms of this information and consent form and to provide the participant with a signed and dated copy.

Name of researcher (please print)	Signature	Date
